# Trends in surgical management of septic arthritis of the knee: a 16-year observational study of 4,809 surgeries in Sweden

**DOI:** 10.1186/s12891-026-09573-8

**Published:** 2026-02-17

**Authors:** Viktor Schmidt, Martin Magnéli, Michael Axenhus

**Affiliations:** 1 Department of Orthopaedic Surgery, Danderyd Hospital, Stockholm, Sweden; 2 Department of Clinical Sciences at Danderyd Hospital, Karolinska Institutet, Stockholm, Sweden

**Keywords:** Debridement, Incidence, Knee, Septic arthritis, Surgery, Sweden

## Abstract

**Introduction:**

Septic arthritis of the knee is a serious condition that requires prompt surgical intervention to prevent irreversible joint damage and systemic complications. Incision and debridement is the standard treatment approach for managing knee septic arthritis. However, limited research exists on long-term trends in these surgical procedures. This study aims to analyse the trends in knee incision and debridement surgeries for septic arthritis in Sweden over a 16-year period (2008–2023), focusing on demographic variations and projections through 2030.

**Methods:**

A retrospective, population-based study was conducted using data from the Swedish National Patient Register (NPR) for the period of January 1, 2008, to December 31, 2023. All patients aged ≥ 15 years who underwent knee incision and debridement surgery for septic arthritis were included. Demographic data were categorized by age, sex, and geographical region. Incidence rates were calculated per 100,000 inhabitants, and Poisson regression models were used to analyse temporal trends and project future surgical incidence rates through 2030.

**Results:**

A total of 4,809 knee incision and debridement surgeries were performed between 2008 and 2023 in Sweden. Men accounted for 3,100 surgeries, compared to 1,709 for women. The highest number of surgeries occurred in 2023 (*n* = 404). The most affected age groups were 65–74 years (1,161 surgeries) and 75–84 years (1,127 surgeries). The overall incidence increased from 3.4 to 4.6 per 100,000 over the study period, with men consistently having higher rates. Significant regional variations were observed, with Jönköping, Jämtland, and Kalmar reporting the highest incidence rates. Projections suggest the incidence will continue to rise, reaching 4.6 per 100,000 by 2030.

**Conclusion:**

The incidence of knee incision and debridement surgeries for septic arthritis in Sweden has steadily increased over the past 16 years, particularly among older adults and men. Significant regional disparities exist, highlighting the need for targeted interventions. Projections suggest that the burden of septic arthritis will continue to rise, necessitating enhanced preventive measures, early diagnosis, and resource planning to meet future healthcare needs.

**Supplementary Information:**

The online version contains supplementary material available at 10.1186/s12891-026-09573-8.

## Introduction

Septic arthritis of the knee is a critical orthopaedic emergency that necessitates urgent surgical intervention to prevent irreversible joint destruction and life-threatening systemic infections [1–3]. The knee joint is the most commonly affected site, although septic arthritis can involve any synovial joint [4–6]. Risk factors include advanced age, diabetes mellitus, immunosuppression, and the presence of prosthetic joints, all of which increase susceptibility to joint infections [7–9]. Men are more frequently affected than women, and factors such as socioeconomic status and ethnicity may influence the incidence of septic arthritis [10–12].

Incision and debridement remain the cornerstone of surgical management, allowing for the removal of infected tissues and reducing bacterial load within the joint [13, 14]. However, there remains a limited body of research analysing trends in this procedure, particularly over extended periods and across large populations. Understanding these trends is essential for identifying at-risk populations, optimizing surgical practises, and improving outcomes.

Furthermore, with the global incidence of septic arthritis increasing—attributed to an aging population and increased prevalence of comorbid conditions such as diabetes—it is essential to forecast future healthcare needs [15]. Such projections can inform policymakers and healthcare providers in planning and implementing preventive strategies.

The primary aim of this study is to provide insights into demographic variations—including sex- and age-specific differences—over the past 16 years in Sweden. The secondary aim is to forecast the future burden of knee debridement surgeries for septic arthritis in Sweden through 2030, providing valuable insights for healthcare planning.

## Materials and methods

### Study design and setting

This retrospective, observational population-based study analyses surgical procedures using data extracted from the Swedish National Patient Register (NPR) between 2008 and 2023. The study follows the RECORD guidelines to ensure methodological transparency and rigor [16]. Data from public healthcare records were analysed to assess surgical trends, patient demographics, and regional variations.

### Healthcare setting

The Swedish National Health Service, administered by the Swedish National Board of Health and Welfare (SNBHW), provides universal healthcare to all citizens, encompassing emergency care, hospital treatments, and outpatient services. While private hospitals exist, septic arthritis surgeries are handled predominantly in public hospitals. Every Swedish resident is assigned a unique personal identification number, which is utilized across all healthcare interactions and integrated into the national health data registers, including the NPR.

### Data source

The NPR is a comprehensive national database that captures all patient interactions within the Swedish healthcare system, encompassing both inpatient and outpatient services since 1964 and 2001, respectively. The database is validated for use in epidemiological studies [17]. Starting in 2021, the registry has shifted from annual to monthly updates, allowing for more accurate tracking of surgical trends. All public and private hospitals in Sweden report surgical procedures, diagnostic codes, and detailed surgical procedure codes using the Nordic Medico-Statistical Committee (NOMESCO) classification system [18]. All orthopaedic departments (*n* = 54) in Sweden are engaged in the NPR. Data regarding surgeries were extracted using the relevant NOMESCO code for knee incision and debridement due to septic arthritis (NGS19). NGS19 involves surgeries performed via an open arthrotomy and arthroscopic technique. It involves opening the joint and debriding infected tissue, usually followed by copious irrigation. Septic arthritis can involve both native and prosthetic joints.

### Inclusion criteria

This study included all individuals aged ≥ 15 years who underwent knee incision and debridement due to septic arthritis, as documented in the NPR between January 1, 2008, and December 31, 2023. All patients with valid Swedish identification numbers were included.

### Exclusion criteria

Patients with revision surgeries, fractures, amputations, and wound revision surgeries were excluded. Patients without a Swedish personal identification number were also excluded.

### Variables

Age was categorized into 10-year intervals, with sex classified as male or female. Regional incidence was determined based on the hospital location where the procedure was performed. Geographic distribution of procedures, patient age, and sex were analysed to identify any trends or disparities.

### Statistical analysis

Incidence rates were calculated per 100,000 inhabitants, with population estimates sourced from Statistics Sweden for each corresponding year. Poisson regression models were used to assess temporal trends and to predict future surgical incidence rates. Incidence rates were stratified by sex, age, and region to capture differences in surgical practices over time. Descriptive statistics were used to summarize patient demographics and surgical details. Confidence intervals (CIs) were computed at a 95% confidence level, and a p-value of < 0.05 was considered statistically significant for any trend analysis.

### Ethics

The data for this study were obtained from publicly available sources and did not include identifiable personal information, making the study exempt from formal ethical review. Informed consent was not required as the data were anonymized and aggregated.

## Results

### Descriptive data and patients

A total of 4,809 incision and debridement surgeries for septic arthritis were performed from 2008 to 2023. Men underwent the majority of surgeries (64%, *n* = 3,100), compared to women (36%, *n* = 1,709). The highest number of surgeries occurred in 2023 (*n* = 404). The most affected age groups are those aged ≥ 65 years, accounting for 57% of all surgeries (*n* = 2,756 ). There was a steady increase in surgeries over time, particularly in older adults, with men consistently undergoing more procedures than women (Table 1, Supplementary Table 1).

### Incidence rates

Overall, the incidence rate of debridement surgeries for septic arthritis increased by 35% during the period 2008 to 2023 (Table 1). Throughout the study period, men consistently exhibited higher incidence rates compared to women and also experienced a greater relative increase (39% vs. 29%) (Table 1).

**Table 1:**
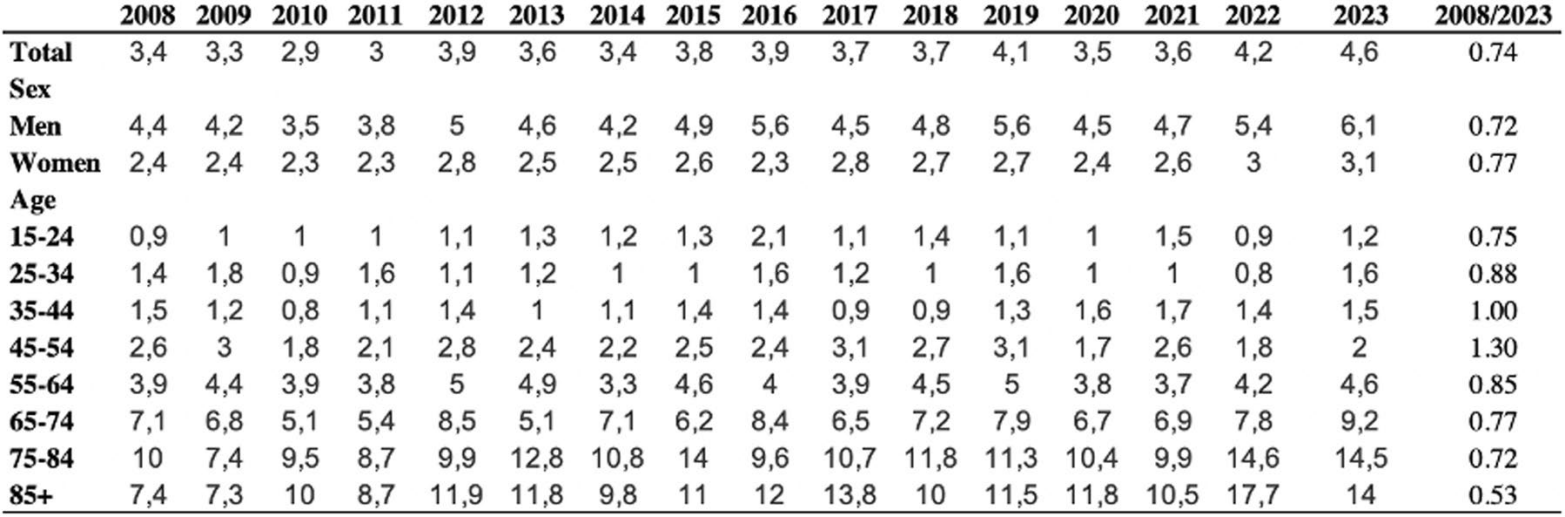
Incidence rates of incision and debridement surgeries for septic arthritis in the knee per 100,000 inhabitants by sex and age group during 2008 to 2023

**Fig. 1:**
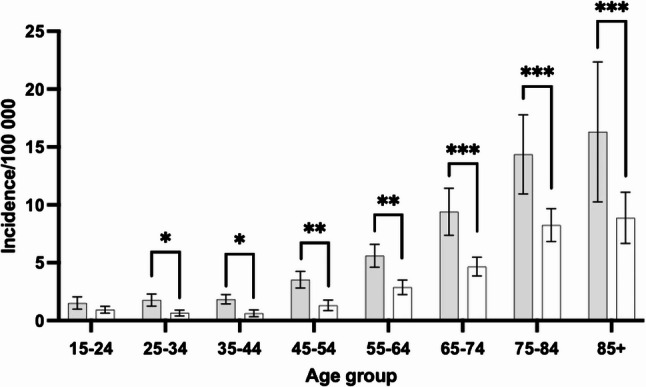
Age-specific annual incidence rates (averages of the years 2008–2023) of incision and debridement surgeries for septic arthritis. Grey bars indicate men and white bars indicate women. *p < 0.05, **p < 0.01, ***p < 0.001

There was a notable increase in surgeries among older individuals, particularly pronounced in the age groups 75–84 years (39% increase) and in those > 85 years (89% increase). In contrast, younger age groups (15–54 years) maintained consistently lower and stable rates throughout the study period (Fig. 1).

### Regional variations

**Fig. 2:**
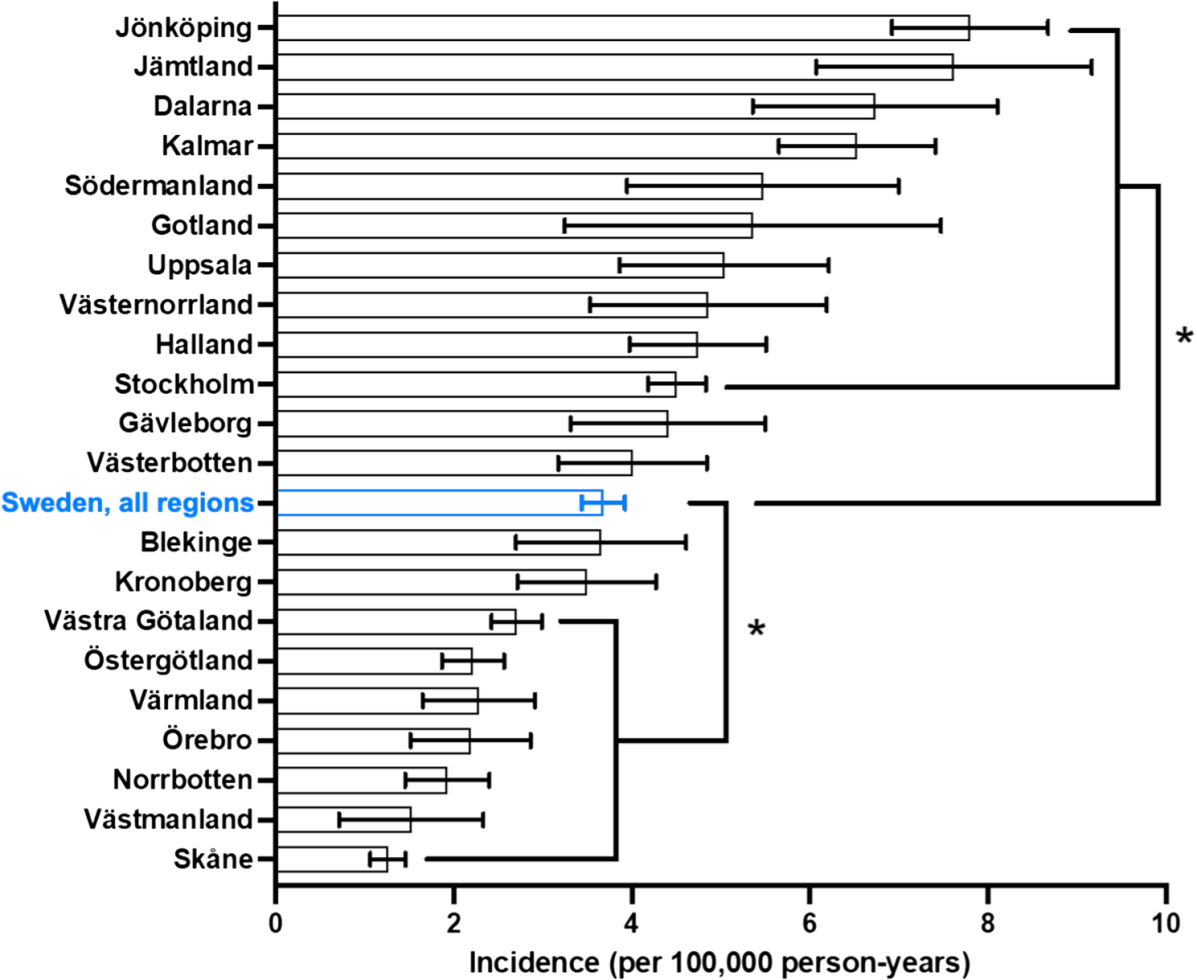
Incidence rates of incision and debridement surgeries for septic arthritis per region during 2008–2023. *p < 0.05 indicates significant difference compared to national average. Error bars indicate 95% confidence interval

Significant regional variation in the incidence of incision and debridement surgeries for septic arthritis was observed across Sweden from 2008 to 2023. The national average incidence rate was 3.7 per 100,000, peaking at 4.6 in 2023, indicating a gradual upward trend. Certain regions, including Jönköping, Jämtland, Dalarna, and Kalmar, consistently reported higher-than-average rates. Jönköping had some of the highest rates, averaging 7.7 per 100,000, and peaking at 10.2 in 2023. Conversely, Skåne and Västmanland showed the lowest incidence rates, with averages around 1.3 per 100,000. In recent years, regions like Södermanland, Gotland, and Uppsala demonstrated notable increases in incidence, suggesting a rising trend in septic arthritis surgeries. These findings underscore the importance of addressing regional disparities in surgical interventions for septic arthritis across Sweden (Fig. 2, Supplemental Table 1).

### Future projections

**Fig. 3:**
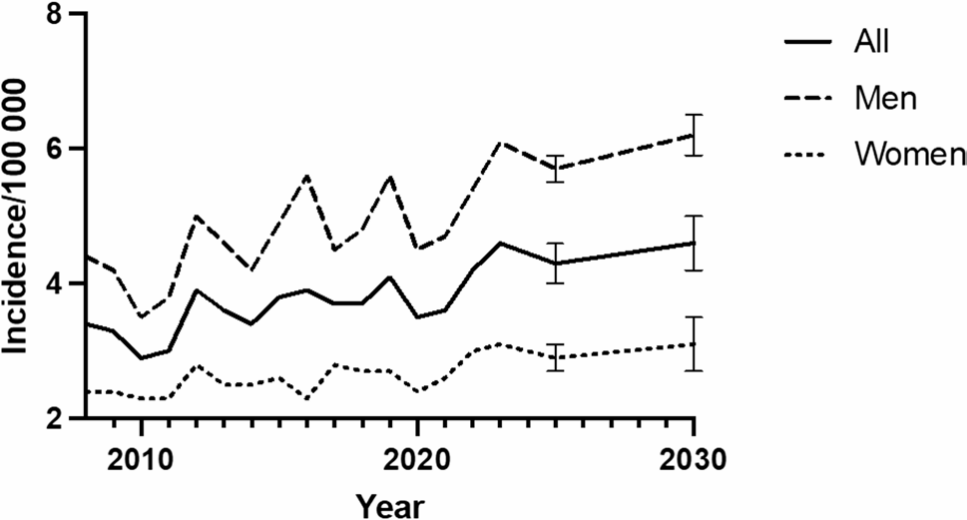
Projected incidence rates of incision and debridement surgeries for septic arthritis in the knee by sex. Error bars indicate 95% confidence interval

The future trend analysis of incision and debridement surgeries for septic arthritis in the knee shows a clear upward trajectory through 2030, based on historical data from 2008 to 2023 and projected incidence rates for 2025 and 2030. The projections for 2025 indicate a slight decrease for the overall population, down to 4.3 per 100,000, with men at 5.7 and women at 2.7. However, by 2030, the overall rate is projected to rise again to 4.6 per 100,000, with men reaching 6.2 and women increasing to 3.5. This forecast suggests that the incidence rate for men will continue to outpace that for women, with the gap potentially widening over time (Fig. 3).

## Discussion

This 16-year observational study shows a steady increase in surgical interventions over time, particularly among older adults and men. Furthermore, significant regional variations emphasize the need for localized healthcare strategies to address disparities in access and treatment.

The slight but consistent rise in the overall incidence of debridement surgeries for knee septic arthritis—from 3.4 to 4.6 per 100,000 over the course of 16 years—indicates the growing burden of this condition. Several factors may contribute to this trend, including the aging population, higher rates of diabetes and other immunocompromising conditions, and an increase in joint replacement surgeries, which are associated with a higher risk of infection [7–9]. These findings highlight the need for proactive monitoring and early intervention to mitigate the impact of septic arthritis, especially in populations at higher risk. Nossent et al. found similar trends with rising incidence of native joint septic arthritis [19].

The increasing incidence of septic arthritis is not unique to Sweden – a population-based study in the United Kingdom similarly showed a significant rise in incidence (approximately 43% increase, from 5.5 to 7.8 per 100,000 between 1998 and 2013) [20], with the greatest increases in older age groups, mirroring our findings. Moreover, the reported incidence rates in the United States are also similar to our results, with an incidence rate of 4–10 per 100,000 [15]. The higher range in other reports may be because the present study only assesses septic arthritis of the knee. These countries have similar aging populations and increasing prevalence of comorbid conditions (e.g. diabetes). In general, many high-income countries are observing similar trends associated with similar risk factors, though the magnitude can differ.

Changes in clinical practice may influence incidence rates – for instance, over the 16-year period, clinicians may have adopted a more proactive approach to suspected septic arthritis, leading to earlier or more frequent surgical washouts, even in borderline cases. This could potentially inflate the surgery counts without an actual increase in true infection incidence. The register does not provide data on false positives and thus we cannot assess this.

Throughout the study period, men consistently exhibited higher rates of knee debridement surgeries than women, a finding consistent with previous literature [10–12, 19, 21]. The incidence among men increased from 4.4 to 6.1 per 100,000, while women’s rates rose more modestly, from 2.4 to 3.1 per 100,000. By the the end of the study period, men were nearly twice as likely as women to experience septic arthritis of the knee. The observed male-to-female ratio was slightly higher than that reported by Morgan et al. [10], about the same as Nossent et al. [19] and approximately half of that reported by Madi et al. [21], confirming the male preponderance. Potential explanations for this gender disparity include higher occupational exposures, differing comorbidity profiles, and variations in healthcare-seeking behaviour.

Additionally, the pronounced increase in surgeries among older age groups—particularly those aged 65–74 and 75–84—highlights the vulnerability of these populations. This supports earlier findings that age is a risk-factor [7, 9]. By 2023, the incidence for the 75–84 age group reached 14.5 per 100,000, and for those aged 85 and older, it peaked at 17.7 per 100,000 in 2022. These trends emphasize the importance of targeted interventions to prevent septic arthritis in high-risk elderly populations.

Significant regional disparities were observed, with areas such as Jönköping, Jämtland, and Dalarna consistently reporting higher-than-average rates of knee debridement surgeries. In contrast, regions like Skåne and Västmanland exhibited much lower incidence rates. These discrepancies could stem from differences in regional healthcare infrastructure, access to specialized care, or variations in infection control practices. Johansson et al. found that 50% of regional variations in health care utilization could be explained by mortality, demographic and socio-economic factors [22]. The observed disparities indicate the need for tailored healthcare strategies that address these regional differences, ensuring that all populations receive timely and effective treatment for septic arthritis.

Our projections suggest a continuing upward trend in knee debridement surgeries through 2030, with the overall incidence expected to rise to 4.6 per 100,000 by that year. The forecasted gap between men and women suggests that men will continue to be disproportionately affected, with an expected incidence of 6.2 per 100,000 for men and 3.5 per 100,000 for women by 2030. These findings underline the growing burden of septic arthritis on healthcare systems, particularly as the population ages. Enhanced preventive measures, such as improved infection control protocols for joint replacements and better management of comorbidities, will be critical in mitigating this rising demand for surgical interventions.

The steady rise in incision and debridement surgeries for knee septic arthritis underscores the importance of heightened vigilance among clinicians, particularly for at-risk groups such as older adults and individuals with underlying health conditions. Early recognition and prompt surgical intervention are essential in preventing severe joint damage and improving patient outcomes. The observed regional disparities further suggest that healthcare systems must adapt to local needs, ensuring equitable access to care across regions. Additionally, the projected increase in surgical demand calls for strategic planning to ensure sufficient surgical capacity and postoperative care resources.

### Limitations

This study has several limitations. First, although we used comprehensive national data, some relevant clinical details—such as infection severity, surgical techniques, and patient-specific risk factors—were not available, potentially limiting the granularity of the analysis. Second, the focus on knee debridement surgeries may restrict the generalizability of our findings to septic arthritis in other joints. Finally, while our future projections are based on robust historical data, they are inherently dependent on assumptions about the continuation of current trends. Changes in healthcare practices or population demographics could alter these future estimates.

## Conclusion

In summary, this study demonstrates a gradual increase in the incidence of knee debridement surgeries for septic arthritis in Sweden, particularly among men and older adults. Regional disparities underscore the need for localized healthcare approaches, while future projections point to an increasing burden on the healthcare system. To effectively manage this growing demand, proactive measures—including improved infection control, early diagnosis, and efficient management of comorbidities—will be essential. Further research is needed to explore the underlying causes of gender and regional disparities and to develop preventive strategies for high-risk populations.

## Supplementary Material

Supplementary Material 1: Supplementary Table 1. Annual incision and debridement surgeries for septic arthritis by sex and age group during 2008 to 2023. Supplemental Table 2. Annual regional incidence of incision and debridement for septic arthritis in the knee during 2008 to 2023.

## Data Availability

The datasets can be obtained from the NPR directly (https://www.socialstyrelsen.se/en/statistics-and-data/statistics/statistical-databases/).
