# Supplementary material for: Trends in surgical management of septic arthritis of the knee: a 16-year observational study of 4,809 surgeries in Sweden
Source: BMC Musculoskelet Disord. 2026 Feb 17;27:173. doi: 10.1186/s12891-026-09573-8 (PMC12930992; doi:10.1186/s12891-026-09573-8)

***Supplementary Table 1:*** *Annual incision and debridement surgeries for septic arthritis by sex and age group during 2008 to 2023.*


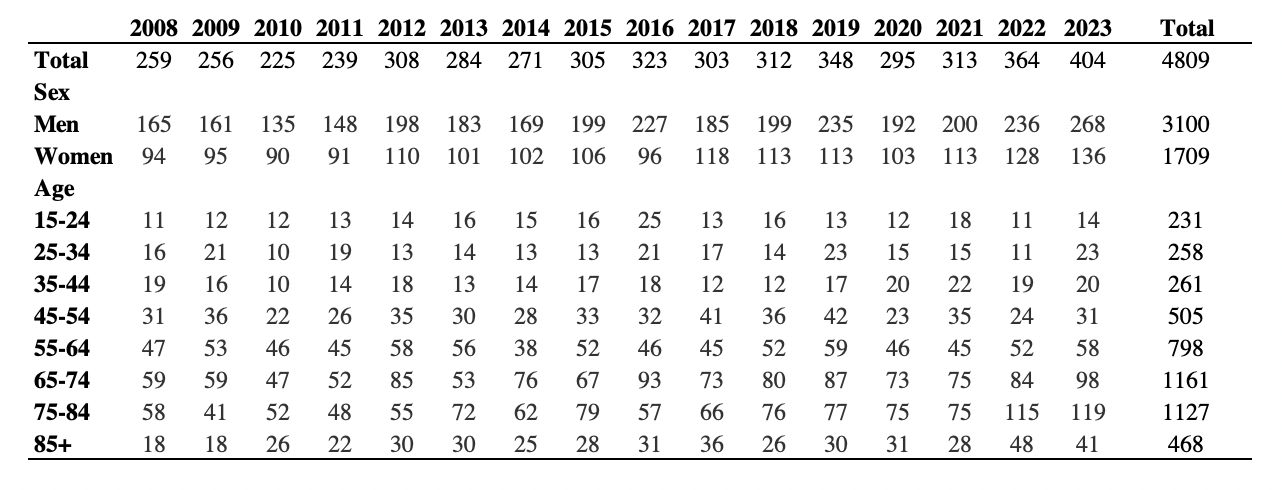


***Supplemental Table 2:****Annual regional incidence of incision and debridement for septic arthritis in the knee during 2008 to 2023.*


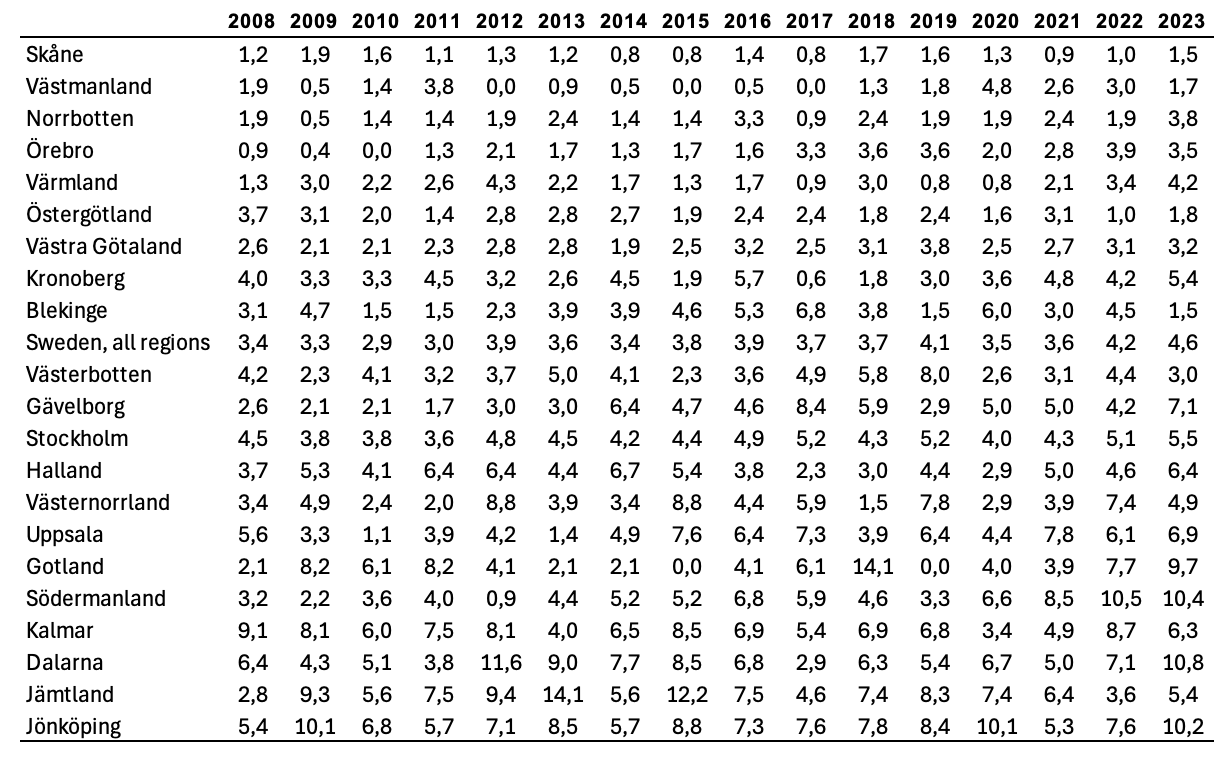

Supplement: Supplementary file 1 — Supplementary Material 1: Supplementary Table 1. Annual incision and debridement surgeries for septic arthritis by sex and age group during 2008 to 2023. Supplemental Table 2. Annual regional incidence of incision and debridement for septic arthritis in the knee during 2008 to 2023. [file 12891_2026_9573_MOESM1_ESM.docx]
